# Bacteremic and non-bacteremic pneumonia caused by *Acinetobacter baumannii* in ICUs of South China: A Clinical and Microbiological Study

**DOI:** 10.1038/s41598-017-13148-y

**Published:** 2017-11-10

**Authors:** Yunfang Tan, Kai Zhou, Xiang Tang, Timothy Kudinha, Luxia Wang, Zhenghui Guo, Murat Akova, Chao Zhuo

**Affiliations:** 1 State Key Laboratory of Respiratory Diseases, the first affiliated hospital of Guangzhou Medical College, Guangzhou, China; 2 State Key Laboratory for Diagnosis and Treatment of Infectious Diseases; Collaborative. Innovation Center for Diagnosis and Treatment of Infectious Diseases, the First Affiliated. Hospital of Medicine School, Zhejiang University, Hangzhou, China; 3 Charles Sturt University, Leeds Parade, Orange, New South Wales Australia; 4 Guangzhou general hospital of Guangzhou Military, Guangzhou, China; 5 Hacettepe University School of Medicine, Department of Infectious Disease, Ankara, 06100 Turkey

## Abstract

*Acinetobacter baumannii* has been a dreadful problem for ICU physicians for a long time. Bacteremic pneumonia (BP) caused by this organism has a higher mortality compared to other organisms. Between 2012 and 2015, 86 BP and 89 non-bacteremic pneumonia (NBP) patients from five ICUs were enrolled into the study. The 7-day and 14-day mortality rates were higher in BP patients than in NBP patients (*P* < 0.001). Procalcitonin elevation, high APACHEII score and recent surgery, were independently associated with BP episodes. Acute respiratory distress syndrome, coma, high APACHEII score and procalcitonin elevation, were independently associated with mortality in the BP group. Extensively drug-resistant isolates were detected in 34.9% of BP and 25.8% of NBP isolates. PFGE identified 12 and 9 genotypes in the BP and NBP isolates, respectively, with 6 genotypes shared by both groups. ST195 was the most prevalent type (40%), followed by ST457 (18.9%). The pandemic clonal complex 92 was predominant, accounting for 94.3% of the strains. For all studied periods, mortality remained higher in the BP than the NBP group. Disease severity was the main risk factor for high mortality in the BP group, and other factors related to mortality were infection, and not treatment or microbiology-related.

## Introduction

The prevalence of health care associated infections caused by *Acinetobacter spp*. is increasing among immunocompromised hosts and patients in intensive care units (ICUs)^1,2^. A large multinational study in Asia (ANSORP) showed that *Acinetobacter spp*. is the most frequently isolated bacterial species (36.5%) in ventilator associated pneumonia (VAP), and the third frequent species (13.6%) in healthcare associated pneumonia (HAP)^3^. In China, *A*. *baumannii* is the most frequently isolated species in both HAP and VAP patients, accounting for 16.2% and 35.7%, respectively^3^. Furthermore, *A*. *baumannii* has a high resistance rate to carbapenems, with a rate of 58.9% in HAP/VAP as per the ANSORP study, and of 53.7% in bloodstream infection, according to China national data^3,4^.Therefore infections caused by this organism present a serious clinical challenge for physicians in health-care settings, resulting in much higher crude mortality rate than other bacterial strains from HAP/VAP^3^.

The relationship between a positive culture of *A*. *baumannii* from lower respiratory tract (LRT) sample and the attributed mortality is less clear in the HAP or VAP patients, as the organism may be considered a colonizer or contaminant, and thus may not reflect the true etiologic pathogen of HAP and VAP. It is very difficult to distinguish between *A*. *baumannii* colonization and infection even if stringent diagnostic procedures, including quantitative culture for LRT samples, are performed^5^.

As reported by ANSORP, the “definite pathogen” of HAP or VAP should be the isolates from blood matching those from culture of the LRT specimens, while the “probable pathogen” and “possible pathogen” are defined as an isolate from LRT culture specimens but with a negative blood culture^3^. In other words, *A*. *baumannii* bacteremia is just the result of pulmonary infection without involvement of other infectious foci in nosocomial settings. In order to establish the causal relationship of bacteria causing pneumonia, such as *P*. *aeruginosa* and *A*. *baumannii*, which have high incidence of colonization in LRT, some authors suggest defining pneumonia caused by a “definite pathogen” as bacteremic pneumonia (BP), and the one caused by a “probable pathogen” or a “possible pathogen” as non-bacteremic pneumonia (NBP)^6,7^.

Numerous studies have investigated the risk factors and mortality of *A. baumannii* bacteremia, with findings consistently suggesting that *A. baumannii* bacteremia has a higher mortality rate than other organisms, ranging from 30% to 60%, especially for imipenem-resistant A. baumannii^6,8^. However, it remains unclear what the attributed mortality of *A. baumannii* bloodstream infections is by different sites of infection. Additionally, there is a scarcity of studies on the microbiological characteristics of *A. baumannii* isolates from bloodstream infections emanating from focal infection.

To date, few studies have comparatively evaluated the clinical features of *A*. *baumannii* pulmonary infection with and without bacteremia, and the genetic differences between strains causing focal and bloodstream infections. The objective of this study was to identify independent risk factors associated with mortality due to *A*. *baumannii* BP and NBP by comparative analysis of clinical characteristics, final outcomes of bacteremic patients, and microbiological features.

## Results

### Clinical Characteristics of *A*. *baumannii* BP and NBP

**Figure 1:**
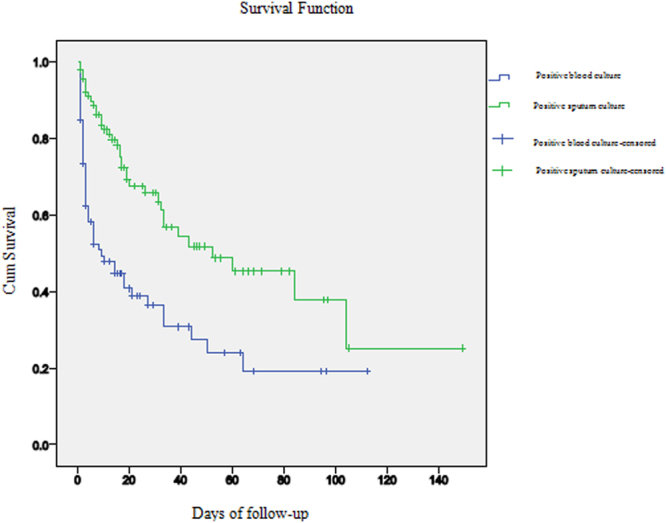
Kaplan–Meier survival curves for patients with NBP and BP.

A total of 175 patients were enrolled in the study, including 86 BP patients and 89 NBP patients. Male patients accounted for 81.4% (70/86) in the BP group and 83.1% (74/89) in the NBP group. There were no significant differences between the two groups in some risk factors of illness (e.g. underlying diseases with chronic pulmonary disease, cerebrovascular disease). However, patients who suffered with ≥ 3 underlying diseases, were more common in the NBP group (51.7% vs 34.9%, P = 0.025). Compared with the NBP group, more BP patients had higher APACHEII scores, acute respiratory distress syndrome (ARDS) and multiple organ dysfunction syndrome (MODS) (P < 0.05). Multivariate Cox regression analysis showed that APACHEII score (OR = 1.048, 95% CI = 1.021–1.076, P = 0.000), PCT elevation (OR = 1.010, 95%CI = 1.005–1.015, P = 0.000), recent surgery (OR = 1.872, 95% CI = 1.211–2.894, P = 0.005), and central venous catheter (OR = 2.334, 95% CI = 1.054–5.169, P = 0.037), were the risk factors for HAP patients complicated with bloodstream infection. The overall mortality rate of BP patients was higher than that of NBP patients (59.3% vs 40.4%, P  =  0.013), especially for 7-day mortality (43.0% vs 13.5%, P < 0.001) and 14-day mortality (48.8% vs 19.1%, P < 0.001) (Table 1). The survival curve for the BP group rapidly declined in the initial five to seven days after the diagnosis, then was on a gradual downward slope from 30 days onwards (Log-rank, X^2^ = 21.975, P < 0.05) (Fig. 1).

**Table 1:** Clinical characteristics of bacteremic and non-bacteremic nosocomial pneumonia patients caused by *Acinetobacter baumannii*.

Variable	BP(86)	NBP(89)	Statistic	P value
	n (%)	n (%)		
age, mean (y)	69.92 ± 14.65	71.63 ± 14.91	t = −0.765	0.445
male gender	70 (81.4)	74 (83.1)	χ² = 0.092	0.762
APACHEII score in admission of ICU	23.31 ± 7.28	20.06 ± 7.66	t = 2.88	0.004
Co-morbidities at admission				
coma	31 (36.0)	26 (29.2)	0.930	0.335
urinary tract infection	1 (1.2)	5 (5.6)	2.622	0.105
wound infection	3 (3.5)	0	3.159	0.076
COPD	25 (29.1)	27 (30.3)	0.034	0.854
cerebral vascular disease	18 (20.9)	27 (30.3)	2.026	0.155
hypertension	38 (44.2)	42 (47.2)	0.159	0.690
diabetes mellitus	20 (23.3)	21 (23.6)	0.003	0.958
ARDS	42 (48.8)	15 (16.9)	20.371	0.000
malignancy	14 (16.3)	23 (25.8)	2.399	0.121
MODS	16 (18.6)	13 (14.6)	0.506	0.477
three or more underlying diseases	30 (34.9)	46 (51.7)	5.026	0.025
length of ICU prior to culture	15.34 ± 25.22	9.46 ± 13.80	Z = −0.681	0.496
length of MV prior to culture	13.16 ± 19.51	9.22 ± 13.998	Z = −1.068	0.286
C-reactive protein	68.28 ± 59.65	90.13 ± 81.79	Z = −1.303	0.193
PCT in admission of ICU	18.42 ± 44.60	3.05 ± 9.66	Z = −4.489	0.000
Invasive procedures				
mechanical ventilation	86 (100)	89 (100)	—	—
recent surgery	38 (44.2)	10 (11.2)	23.856	0.000
CRRT	33 (38.4)	27 (31.5)	1.253	0.263
central venous catheter	77 (89.5)	67 (75.3)	6.096	0.014
foley catheter	69 (80.2)	77 (86.5)	1.249	0.264
nasogastric tube	80 (93.0)	78 (87.6)	1.445	0.229
Previous antibiotic used (within 1 month)				
sulbactam+ β-lactams	38 (44.2)	27 (30.3)	3.593	0.058
carbapenems	65 (75.6)	55 (61.8)	3.856	0.050
fluoroquinolones	19 (22.1)	21 (23.6)	0.056	0.813
linezolid	9 (10.5)	5 (5.6)	1.396	0.237
glycopeptides	43 (50.0)	28 (31.5)	6.235	0.013
cephalosporin	11 (12.8)	8 (9.0)	0.653	0.419
amikacin	3 (3.5)	1 (1.1)	1.095	0.295
tigecycline	4 (4.7)	3 (3.4)	0.187	0.666
used 3 or more antibiotics	38 (44.2)	28 (31.5)	3.015	0.082
proportion of XDR strains	30 (34.9)	23 (25.8)	1.693	0.193
Outcome				
duration of ICU	26.43 ± 33.16	33.01 ± 34.83	Z = −2.690	0.007
duration of MV	25.97 ± 31.69	32.16 ± 35.73	Z = −2.369	0.018
Survival ≥30 d	17 (19.8)	32 (36.0)	5.685	0.017
in-hospital mortality	51 (59.3)	36 (40.4)	6.218	0.013
7-day mortality	37 (43.0)	12 (13.5)	18.931	0.000
14-day mortality	42 (48.8)	17 (19.1)	17.305	0.000
28-day mortality	46 (53.5)	26 (29.2)	4.778	0.029
XDR mortality	18 (20.9)	14 (15.7)	0.791	0.374
MDR mortality	31 (36.0)	22 (24.7)	2.658	0.103
days of death after culture	8.92 ± 13.73	21.47 ± 23.31	Z = −3.870	0.000

Data presented as mean  ±  standard deviation or n (%); BP, bacteremic pneumonia; NBP, non-bacteremic pneumonia; APACHE, acute physiology and chronic health evaluation; COPD, chronic obstructive pulmonary disease; ARDS, Acute respiratory distress syndrome; MODS, multiple organ dysfunction syndrome; ICU, intensive care units; MV, mechanical ventilation; PCT, procalcitonin; CRRT, continuous renal replacement therapy; XDR, extensively drug-resistant; MDR, multi-drug resistance.

The clinical characteristics of survivors and non-survivors in the BP group were also studied. The incidence of ARDS (66.7%) and coma (45.1%) in the non-survival group was higher than in the survival group (P < 0.05). Furthermore, the APACHEII score (25.31 ± 7.28) and PCT elevation (26.07 ± 56.32) were higher in the non-survival group than the survival group (P < 0.05). Multivariate Cox regression analysis showed that the high APACHE II score (OR for death = 1.058, 95%CI = 1.022–1.096, P = 0.002) and ARDS (OR for death = 2.229, 95%CI = 1.222–4.065, P = 0.009), coma (OR for death = 2.000, 95%CI = 1.084–3.689, P = 0.027) and PCT elevation (OR for death = 1.007, 95%CI = 1.001 to 1.013, P = 0.025, were independently associated with mortality of the BP group (Table 2).

**Table 2:** Clinical characteristics of survivors and non-survivors in the BP group.

Variable	Survivors (35)	non-survivors (51)	Statistic	P value
	n (%)	n (%)		
male gender	25 (71.4)	45 (88.2)	3.872	0.049
age, mean(y)	67.29 ± 10.64	71.73 ± 16.72	t = −1.504	0.136
length of stay ICU, mean d	30.51 ± 31.659	23.63 ± 34.175	z = −1.579	0.114
length of ICU prior to bacteremia	17.34 ± 22.643	16.22 ± 23.466	Z = −0.004	0.996
mechanical ventilation	35 (100)	51 (100)	—	—
length of MV, mean d	32.71 ± 34.335	21.33 ± 29.203	z = −1.958	0.050
length of MV prior to bacteremia	18.23 ± 22.196	12.67 ± 22.924	z = −2.105	0.035
Co-morbidities at admission				
hypoproteinemia	29 (82.9)	44 (86.3)	0.189	0.664
ARDS	8 (22.9)	34 (66.7)	15.943	0.000
coma	8 (22.9)	23 (45.1)	4.454	0.035
wound infection	1 (2.9)	2 (3.9)	0.070	0.792
urinary tractin fection	1 (2.9)	0	1.474	0.225
COPD	9 (25.7)	16 (31.4)	0.322	0.570
cerebral vascular disease	5 (14.3)	13 (25.5)	1.574	0.210
hypertension	16 (45.7)	22 (43.1)	0.056	0.813
diabetes mellitus	8 (22.9)	12 (23.5)	0.005	0.942
cardiac disorders	6 (17.1)	16 (31.4)	2.208	0.137
MODS	6 (17.1)	10 (19.6)	0.083	0.773
malignancy	6 (17.1)	8 (15.7)	0.032	0.857
3 or more underlying diseases	14 (40.0)	16 (31.4)	0.680	0.410
Invasive procedures				
CRRT	13 (37.1)	20 (39.2)	0.038	0.846
central venous catheter	32 (91.4)	45 (88.2)	0.226	0.635
foley catheter	28 (80.0)	41 (80.4)	0.002	0.964
nasogastric tube	34 (97.1)	46 (90.2)	1.543	0.214
recent surgery	17 (48.6)	21 (41.2)	0.460	0.498
Laboratory parameters				
APACHE II score	20.40 ± 6.33	25.31 ± 7.28	Z = −3.076	0.002
C-reactive protein	64.34 ± 61.14	73.18 ± 55.65	Z = −1.174	0.241
PCT	7.267 ± 9.65	26.07 ± 56.32	Z = −2.335	0.023
proportion of XDR strains	12 (34.3)	18 (35.3)	0.009	0.923
Previous antibiotic used (within 1 month)				
sulbactam+ β-lactams	16 (45.7)	22 (43.1)	0.056	0.813
carbapenems	28 (80.0)	37 (72.5)	0.624	0.429
tigecycline	2 (5.7)	2 (3.9)	0.150	0.698
linezolid	4 (11.4)	5 (9.8)	0.058	0.809
fluoroquinolones	10 (28.6)	9 (17.6)	1.439	0.230
glycopeptides	19 (54.3)	24 (47.1)	0.434	0.510
cephalosporin	4 (11.4)	7 (13.7)	0.098	0.754
amikacin	1 (2.9)	2 (3.9)	0.070	0.792
used 3or more antibiotics	17 (48.6)	21 (41.2)	0.460	0.498
Antimicrobial therapy in the occurrence of BP				
sulbactam+ β-lactams	15 (42.9)	10 (19.6)	5.441	0.020
carbapenems	25 (71.4)	44 (86.3)	2.884	0.089
tigecycline	6 (17.1)	5 (9.8)	1.002	0.317
aminoglycosides	2 (5.7)	1 (2.0)	0.869	0.351
fluoroquinolones	3 (8.6)	5 (9.8)	0.037	0.847
Combination antimicrobial therapy				
tigecycline+sulbactam/β-lactam	3 (8.6)	2 (3.9)	0.820	0.365
carbapenems+sulbactam/β-lactams	9 (25.7)	8 (15.7)	1.316	0.251
appropriate antimicrobial therapy	14 (40.0)	12 (23.5)	2.670	0.102

Data presented as mean ± standard deviation or n (%); ICU, intensive care units; MV, mechanical ventilation; ARDS, acute respiratory distress syndrome; COPD, chronic obstructive pulmonary disease ; MODS, multiple organ dysfunction syndrome; CRRT, continuous renal replacement therapy; APACHE, acute physiology and chronic health evaluation; PCT, procalcitonin; XDR, extensively drug-resistant.

### Microbiological Studies

Non-repetitive clinical isolates (n = 261) including 172 paired isolates obtained from the BP group (one each from LRT sample and blood culture in each patient) and 89 from NBP group, were identified as *ACB complex* by the *API 20 NE* system (bioMérieux Vitek, Marcy l'Etoile, France) . Using the ITS region sequence of the 16S-23S rRNA gene, BP group isolates were discriminated into 3 groups, namely *A*. *baumannii* (n = 170), *genomic species 3* (n = 1), and *genomic species 13TU* (n = 1), whilst in the NBP group, isolates were classified as *A*. *baumannii* (n = 87), *genomic species 3* (n = 1), and *genomic species 13TU* (n = 1). *A*. *baumannii* was the predominant type in both patient groups, accounting for 98.5% of the isolates.

Strains of the BP and NBP groups showed similar antimicrobial resistant profiles. In total, 95.8% of the tested strains were resistant to meropenem, with MIC_90_ ≥128 µg/ml. The prevalence of extensively drug resistant *A*. *baumannii* (XDRAB) was 34.9% in the BP group and 25.8% in the NBP, as well as 34.3% (12/35) in survivors and 35.3% (18/51) in non-survivors in the BP group. The XDRAB isolates had higher susceptibility rates to polymyxin B (96.6%) with MIC_90_ of 2 µg/ml, and tigecycline (56.6%) with MIC_90_ of 4 µg/ml (Tables 3 and 4).

**Table 3:** Comparison of antimicrobial susceptibilities of *Acinetobacter baumannii* from BP and NBP groups.

Antimicrobial agent	BP		NBP		X²	P value
	R%	MIC_50_/MIC_90_	R%	MIC_50_/MIC_90_		
cefepime	95.35	64/≥128	92.13	64/≥128	0.767	0.381
ceftriaxone	96.51	≥128/≥128	91.01	≥128/≥128	2.246	0.134
ciprofloxacin	95.35	≥16/≥16	95.51	≥16/≥16	0.002	0.960
imipenem	95.35	32/≥128	91.01	16/≥128	1.288	0.256
piperacillin/tazobactam	96.51	≥512/4/≥512/4	89.89	256/4/≥512/4	3.005	0.083
gentamicin	89.53	32/≥128	93.26	32/≥128	0.774	0.379
tobramycin	87.21	32/≥128	86.52	32/≥128	0.018	0.892
levofloxacin	55.81	8/≥32	56.18	8/≥32	0.002	0.961
tigecycline	22.09	1/4	15.73	1/4	1.157	0.282
cefperazone/sulbactam	87.21	64/≥256	84.27	64/≥256	0.309	0.579
minocyline	51.28	8/32	53.85	16/32	1.799	0.180
amikacin	65.63	128/≥512	69.64	128/≥512	0.152	0.697
ceftazidime	96.51	≥128/≥128	95.51	≥128/≥128	0.115	0.734
ampicillin/sulbactam	94.19	128/≥512	95.51	128/≥512	0.156	0.693
meropenem	95.35	≥32/≥32	93.26	16/≥32	0.355	0.551
polymyxin B	3.49	2/2	3.37	2/2	0.002	0.966

**Table 4:** Comparison of antimicrobial susceptibilities of blood isolates in survivors and non-survivors.

Antimicrobial agent	Survivors		non-survivors		X²	P value
	R%	MIC_50_/MIC_90_	R%	MIC_50_/MIC_90_		
cefepime	94.29	64/≥128	96.08	64/≥128	0.150	0.698
ceftriaxone	97.14	≥128/≥128	96.08	≥128/≥128	0.070	0.792
ciprofloxacin	94.29	≥16/≥16	96.08	≥16/≥16	0.150	0.698
imipenem	94.29	32/≥128	96.08	32/≥128	0.150	0.698
piperacillin/tazobactam	97.14	≥512/4/≥512/4	96.08	≥512/4/≥512/4	0.070	0.792
gentamicin	88.57	32/≥128	90.20	32/≥128	0.537	0.464
tobramycin	82.86	32/≥128	90.20	32/≥128	1.202	0.273
levofloxacin	57.14	8/≥32	54.90	8/≥32	0.042	0.837
tigecycline	20.00	1/4	23.53	1/4	0.150	0.698
cefperazone/sulbactam	82.86	64/128	90.20	64/≥256	1.002	0.317
minocyline	47.37	8/16	55.00	16/32	0.227	0.634
amikacin	64.71	128/≥512	66.67	128/≥512	0.014	0.907
ceftazidime	94.29	64/≥128	98.04	≥128/≥128	0.869	0.351
ampicillin/sulbactam	91.43	128/256	96.07	128/≥512	0.820	0.365
meropenem	94.29	≥32/≥32	96.08	≥32/≥32	0.150	0.698
polymyxin B	0	2/2	5.88	2/2	2.133	0.144

### PFGE Analysis

The 172 strains from the BP group were classified into 12 genotypes. Seven major types accounted for 62.8% of the isolates, including types A (n = 24), B (n = 20), C (n = 18), D (n = 14), E (n = 12), F (n = 12), and G (n = 8). The remaining 5 types (H, I, J, K, and L) each consisted of 2–4 strains. Isolates from blood and LRT sample of the same patient belonged to the same genotype. Eighty-six strains of the NBP group were classified into 9 genotypes. For the BP group, 69 strains were classified into six clones (A–F), and the remaining 20 strains belonged to 3 types (M, N, and Q).

### MLST Analysis

**Figure 2:**
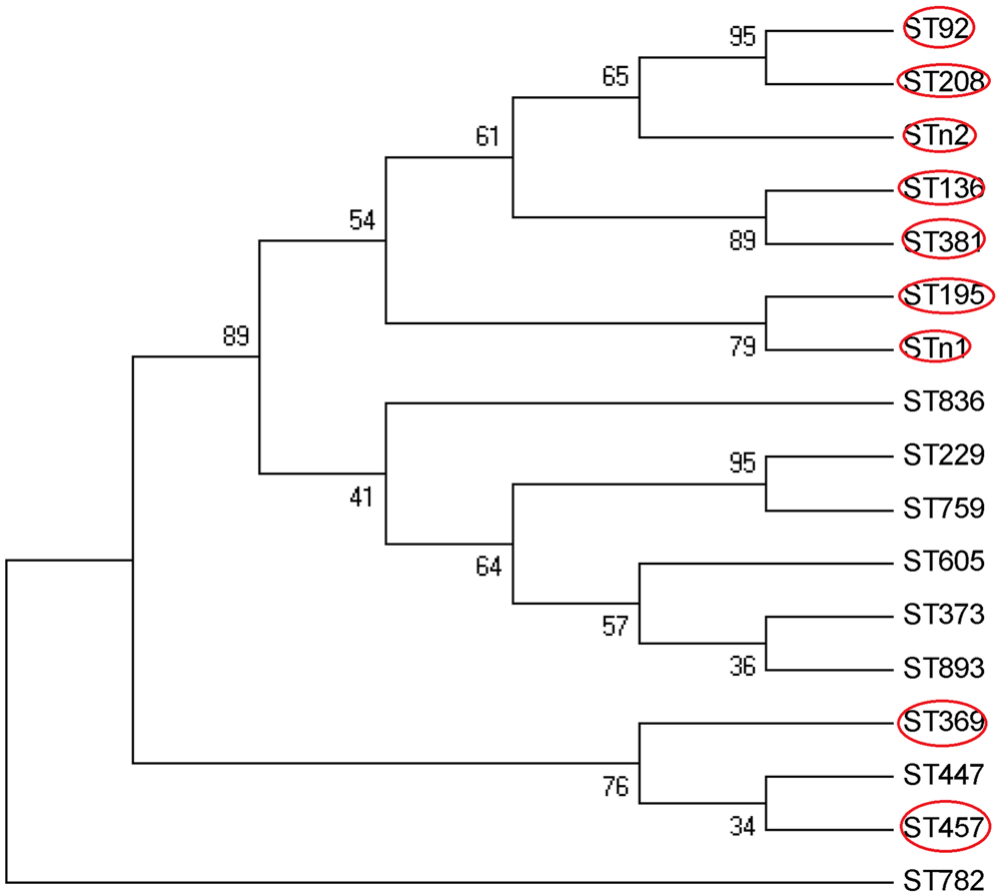
Genetic relatedness of STs detected in 175 strains. Members of CC92 are indicated in red circles.

The blood culture isolates of the BP group (n = 86) and all isolates of the NBP group (n = 89) were further typed by MLST. The isolates were assigned to 16 STs, including 2 new STs (STn1 and STn2). The prevalence of ST195, ST457, ST208 and STn1 (1-15-3-2-2-96-3) (a variant of ST457 with one-allele difference) was 40%, 18.9%, 13.1% and 12%, respectively. The five STs had similar distribution in the BP and NBP groups, as well as in the survival and non-survival BP group patients (Table 5). eBURST analysis revealed that eight of 16 STs (ST136, ST195, ST208, ST457, ST369, ST381, STn1, STn2) belonged to the clonal complex 92 (CC92) as shown in Fig. 2, accounting for 94.3% in total.

**Table 5:** MLST analysis of strains from bacteremic and non-bacteremic nosocomial pneumonia patients caused by *Acinetobacter baumannii*.

STs	BP(86)	NBP(89)	X²	P value	non-survivors(51)	Survivors(35)	X²	P
	n (%)	n (%)			n (%)	n (%)		
136	5 (5.81)	5 (5.62)	0.003	0.955	2 (3.92)	3 (8.57)	0.820	0.365
195	36 (41.86)	34 (38.20)	0.244	0.621	22 (43.14)	14 (40.00)	0.084	0.772
208	7 (8.14)	16 (17.98)	3.708	0.054	4 (7.84)	3 (8.57)	0.015	0.903
229	1 (1.16)	1 (1.12)	0.001	0.981	0	1 (2.86)	1.474	0.225
369	2 (2.33)	1 (1.12)	0.375	0.54	0	2 (5.71)	2.984	0.084
373	1 (1.16)	0	1.041	0.308	1 (1.96)	0	0.694	0.405
381	0	3 (3.37)	2.949	0.086	0	0	—	—
447	1 (1.16)	0	1.041	0.308	0	1 (2.86)	1.474	0.225
457	19 (22.09)	14 (15.73)	1.157	0.282	13 (25.49)	6 (17.14)	0.84	0.359
605	1 (1.16)	0	1.041	0.308	0	1 (2.86)	1.474	0.225
759	0	1 (1.12)	0.972	0.324	0	0	—	—
782	0	1 (1.12)	0.972	0.324	0	0	—	—
836	1 (1.16)	1 (1.12)	0.001	0.981	1 (1.96)	0	0.694	0.405
893	0	1 (1.12)	0.972	0.324	0	0	—	—
n1	11 (12.79)	10 (11.24)	0.1	0.752	8 (15.69)	3 (8.57)	0.942	0.332
n2	1 (1.16)	1 (1.12)	0.001	0.981	0	1 (2.86)	1.474	0.225

MLST analysis of strains obtained from survivors and non-survivors among BP group.

## Discussion

*A*. *baumannii* infections can cause high mortality varying from 20% to 70% of nosocomial infections^3,9^. The difference in the mortality rate depends on several factors including type of microorganism involved, host immunity, severity of illness, and likelihood of receiving antibiotic treatment. Consequently, it is difficult to evaluate infection-related mortality caused by *A*. *baumannii* using overall mortality or in-hospital mortality. This study found that BP patients were more prone to death than NBP patients in the shorter duration time period (7 days), with 88.1% (37/42) of BP patient deaths occurring during this period, suggesting that 7-day mortality was a better predictor of patient outcome in *A*. *baumannii* BP than 28-day or overall mortality. However, the patients took a turn for the worse when some common risk factors, such as underlying disease, long-term hospitalization, and invasive examination, were taken into consideration. These risk factors also worsened the outcome in 28-day mortality of NBP patients. Mortality remained significantly higher in the BP group for all recorded periods, including 28 days after infection.

Consistent with previous studies, ARDS, coma, high PCT and high APACHEII score, were the risk factors of death in the BP group^10–12^. However, no association was found between receiving an appropriate antibiotic treatment and the mortality rate. The reasons for this could be: (i) the high prevalence of XDRAB strains in our study collection presented a great challenge for the antimicrobial treatment. According to ESCMID guidelines, the present regimen in the treatment of XDRAB infection, including colistin, tigecycline, and sulbactam, has limited clinical evidence for successful treatment^13^. Therefore, at the present moment, the recommended antimicrobial therapy for XDRAB is not appropriate as it is not supported by clinical evidence. (ii) due to the restriction on certain doses of tigecycline in China, it is difficult to follow the recommended drug treatment dosage guidelines or expert consensus on XDRAB^14^. For example, the specified tigecycline daily dosage in China is no more than 100 mg/day, which is an insufficient dose for pneumonia or bacteremia as per guidelines. If the MIC is >1 μg/mL, a higher dosage schedule (75 or 100 mg every 12 hours, after a loading dose of 150 or 200 mg, respectively) is required^14^. Since 43.6% of the strains in this study had MIC >1 µg/ml, it means that treatment failure may have occurred in <50% of patients using tigecycline at 100 mg/d^15^. (iii) timely initiation of appropriate therapy is a key point for BP patients due to the severity and rapid development of the disease in this group. However, in this study, positive culture results were usually obtained after 2 or 3 days, which delayed the best treatment time point. Taken together, our results imply that fully assessing the dynamic risk factors of BP, early detection of pathogens, and timely initiation of appropriate antibiotics, may be the key measures to improve the prognosis of BP patients^13^.

Interestingly, findings from this study suggest that patients who undergo surgery may easily develop BP in comparison to the control group. This may be caused by endotracheal intubation, mechanical ventilation, residual effects of anesthetics, and insufficient coughing due to postoperative wound pain, acquired by surgical patients, resulting in colonizing strains accessing lung tissue. It is necessary to give early passive surveillance and decolonization in management of such patient groups^5^.

Most studies on *A*. *baumannii* are concentrated on clinical features, and there are very few studies on the microbiologic characteristics that promote the progression of the organism from focal to bloodstream infection. Most studies focused on the comparison of carbapenem resistant and carbapenem susceptible isolates, and the association of *Acinetobacter* genomic species with clinical outcomes^13,16^. In the present study, the proportion of carbapenem-resistant isolates did not differ significantly between the BP and NBP groups. Likewise, in the BP group, there was no difference in the proportion of individuals in the death and survivor groups. Additionally, 98.5% of the tested strains belonged to *A*. *baumannii*, which made it difficult to compare the effect of bacterial phenotype on relative clinical risk between the two groups. Furthermore, there was no significant difference in the ST distribution of strains in the two groups, and 94.3% strains belonged to CC92, the most widely distributed population in the world^17^. Notably, CC92 is frequently associated with MDR or XDR profile, and is responsible for more treatment failures than other clones when empirical therapy is used. This is especially an issue in China, where other drugs rather than colistin or tigecycline, are used in treatment, and which according to previous studies, explains the increase in indirect mortality in CC92 epidemic areas^18,19^. Although all patients in the present study were treated with polymyxin, which was used in combination therapy, in 32.4% (n = 30) of the patients, the 30-day mortality rate was still 83.7%. Multivariate analysis revealed that septic shock at diagnosis of XDR-ABC infection was a risk factor for 30-day mortality, which is in agreement to a previous study^20^. Therefore, early detection of high-risk clones may improve the prognosis of patients.

There are some limitations in this study. Lack of genome sequences of some predominant clones such as ST195 and ST 457 in the BP group is a limitation. Such data could provide extra information to explore the association of genetic characteristics with the occurrence of BP. Notably, ST457 is a predominant type found in almost all patients with bacteremic MDRAB since being described in 2013 in Hong Kong^21^. To explore the virulence of the emerging clone, we further sequenced the genome of a ST457 strain XH860, and obtained a complete genome (GeneBank accession no. CP014538). Comparison of virulence factors (VFs) between XH860 and the recently published hypervirulent ST10 strain LAC_4 (GeneBank accession no. CP007712) by blasting against VFDB, revealed no difference on the major VFs studied. This suggests that ST457 would also be a hypervirulent clone as ST10. Additionally, some VFs involved in type VI secretion system and quorum sensing were found in XH860 but not in LAC_4. The difference in the distribution of VFs in specific clones needs to be investigated further.

In conclusion, this study found that mortality remained significantly higher in the BP group than the NBP group for all recorded periods, including 28 days of infection, but was especially marked for the 7-day and 14-day mortality. Disease severity was the main risk factor for the high mortality rate in the BP group, and other factors related to mortality in this group were infection related, and not treatment-or microbiology-related.

## Methods

### Study Design and Inclusion Criteria

This study was carried out from July 2012 to July 2015, and included three tertiary hospitals in South China. All enrolled patients were admitted for a diagnosis of HAP/VAP, and had a positive culture of *A*. *baumannii* from lower respiratory tract (LRT), and/or blood culture, according to the American Thoracic Society/Infectious Disease Society of America guidelines^22^. *A*. *baumannii* bacteremic pneumonia (BP) was defined as isolation of *A*. *baumannii* from at least one blood culture and one LRT sample culture in the same HAP/VAP patient within 48 hours^6^. Furthermore, the positive blood culture of *A*. *baumannii* was not related to another source of infection, such as catheter-related bacteremia. Non bacteremic pneumonia (NBP) was defined as only the isolation of *A*. *baumannii* in LRT sample, and with a negative blood culture from HAP/VAP patients, within 48 hours. Furthermore, organisms other than *A*. *baumannii*, detected in both respiratory and blood cultures within this period, were defined as inconsistent microbiology, and such cases were excluded from the study.

### Collection of Clinical Data

Clinical data covering demography, risk factors, clinical characteristics, and clinical outcomes, were collected. Data collected included demographic data (gender, age, hospitalization time, ICU stay time etc.); underlying diseases (such as chronic obstructive pulmonary disease, cerebrovascular disease, diabetes, cardiovascular disorders, surgery within 30 days etc.); vital signs (body temperature, heart rate, respiratory rate, blood pressure, oxygenation index); lab examination (white blood cell count, platelet, C-reactive protein, procalcitonin); and other clinical characteristics (APACHE score, invasive operation (such as continuous renal replacement therapy and the central venous catheter, endotracheal intubation or cut channel catheter, gastrointestinal nutrition tube, etc.)), at admission in ICU and within 24 hours of the first positive blood culture or sputum culture. And finally, the use of antibiotics within 30 days before the first positive blood culture or sputum culture of *A*. *baumannii*, was recorded. The use of antibiotics for BP and NBP, the proportion of appropriate antibiotic therapy; in-hospital mortality, 7-day, 14-day and 28-day mortality, were documented. The 7-day (14- and 28-day) mortality was defined as death occurring ≤7 days (14 and 28 days) after blood cultures were positive for *A*. *baumannii*.

Appropriate antimicrobial therapy was defined as administration of ≥1 antimicrobial agent to which the causative pathogen was susceptible *in vitro*, within 48 hours after the onset of BP and NBP, and with an approved route and dosage appropriate for end organ function^23^. Due to the unavailability of polymyxin B and/or colistin in China, antimicrobial agents including carbapenem, sulbactam, or its fixed-dose combinations with ampicillin or cefoperazone, and tigecycline, may be selected for combination therapy at a higher dosage, if *A*. *baumannii* isolates show intermediate or minimum inhibitory concentrations (MIC) values closer to the breakpoints of susceptibility. These combination therapies are also considered as appropriate antimicrobial therapy^24^.

### Microbiological Studies

All the LRT samples including sputum, broncho-alveolar lavage, and tracheal aspirates, were collected according to microbiological standard methods of HAP/VAP guidelines^3^. Non-repetitive clinical isolates were identified as *ACB complex* by the *API 20 NE* system (*bioMérieux Vitek*, *Marcy l'Etoile*, *France*) .Since biochemical classification tests cannot distinguish among *Acinetobacter calcoaceticus*, *Acinetobacter baumannii*, *Acinetobacter* genomic species 3 and *Acinetobacter genomic species 13TU*, all the types were referred to as *A*. *Calcoaceticus*-*A*. *baumannii* complex (ACB complex) in the routine work. We use the 16S-23S rRNA gene ITS region sequencing to identify *ACB complex*. Reference strains of *Acinetobacter calcoaceticus*, *Acinetobacter genomic species* 3 and *Acinetobacter genomic species 13TU*, were kindly provided by Fu Y^25^.

The MIC of each antimicrobial agent was determined by the broth microdilution method, and results were interpreted according to the criteria of the CLSI for broth dilution^18^. Extensively drug resistant (XDR) was defined as non-susceptibility to at least one agent in all but two or less antimicrobial categories (i.e. bacterial isolates remain susceptible to only one or two categories) according to European expert consensus^26^.

### Genetic Relatedness of Tested Strains

Pulsed-field gel electrophoresis (PFGE) analysis of *Apa I*-digested genomic DNA was performed to determine the genetic relatedness of *A*. *baumannii* isolates using a CHEF-Mapper XA System (Bio-Rad Laboratories, Hercules, CA, USA) as described previously^27^. The interpreting criteria used was described by Tenover^28^, combining unweighted pair group method with hierarchic averages (UPGMA). Isolates were assigned the same pulsotype if the value of Dice coefficient of similarity was >80%.

Multilocus sequence typing (MLST) was conducted as previously described^29^, based on seven housekeeping genes (*gltA*, *gyrB*, *gdhB*, *recA*, *cpn60*, *gpi*, *rpoD*). The STs were assigned according to *A*. *baumannii* MLST database (http://pubmlst.org/abaumannii/). The clonal complexes (CCs) were analyzed by the Based upon Related Sequence Types (BURST) clustering algorithm (eburst.mlst.net).

### Statistical Analysis

Statistical analysis was performed using SPSS version 22.0. The dichotomous variables were analyzed by chi-square test or Fisher’s exact test, whereas the continuous variables were analyzed by independent t-test. To construct the survival curve, we used the Kaplan-Meier method, calculating *P* values by the log-rank test. Multivariate analysis was performed by stepwise logistic regression. Results of *in vitro* susceptibility were analyzed using WHONET5.6 software.

### Notes

#### Ethics statement

The participating centers either received ethical approval from the Ethics Committees of their respective hospitals (The First Affiliated Hospital of Guangzhou Medical University, Guangzhou General hospital of Guangzhou military and The first People's Hospital of Foshan), or ethical approval was waived due to the observational nature of the study. All subjects signed written informed consent prior to the study. Patient information was anonymized and de-identified prior to analysis. All methods were carried out in accordance with relevant guidelines and regulations.
